# Paradoxical reactions in Buruli ulcer after initiation of antibiotic therapy: Relationship to bacterial load

**DOI:** 10.1371/journal.pntd.0007689

**Published:** 2019-08-26

**Authors:** Michael Frimpong, Bernadette Agbavor, Mabel Sarpong Duah, Aloysius Loglo, Francisca N. Sarpong, Justice Boakye-Appiah, Kabiru M. Abass, Mathias Dongyele, George Amofa, Wilson Tuah, Margaret Frempong, Yaw A. Amoako, Mark Wansbrough-Jones, Richard O. Phillips

**Affiliations:** 1 Kumasi Centre for Collaborative Research in Tropical Medicine (KCCR), Kwame Nkrumah University of Science and Technology (KNUST), Kumasi, Ghana; 2 School of Biosciences and Medicine, University of Surrey, Guildford, United Kingdom; 3 Institute of Infection and Immunity, St George’s University of London, United Kingdom; 4 Agogo Presbyterian Hospital, Agogo, Ghana; 5 Tepa Government Hospital, Tepa, Ghana; 6 Dunkwa Government Hospital, Dunkwa-on-Offin, Ghana; 7 Nkawie-Toase Government Hospital, Nkawie, Ghana; 8 School of Medical Sciences, Kwame Nkrumah University of Science and Technology (KNUST), Kumasi, Ghana; Stanford University, UNITED STATES

## Abstract

**Background:**

We investigated the relationship between bacterial load in Buruli ulcer (BU) lesions and the development of paradoxical reaction following initiation of antibiotic treatment.

**Methods:**

This was a longitudinal study involving BU patients from June 2013 to June 2017. Fine needle aspirates (FNA) and swab samples were obtained to establish the diagnosis of BU by PCR. Additional samples were obtained at baseline, during and after treatment (if the lesion had not healed) for microscopy, culture and combined 16S rRNA reverse transcriptase/ IS*2404* qPCR assay. Patients were followed up at regular intervals until complete healing.

**Results:**

Forty-seven of 354 patients (13%) with PCR confirmed BU had a PR, occurring between 2 and 42 (median 6) weeks after treatment initiation. The bacterial load, the proportion of patients with positive *M*. *ulcerans* culture (15/34 (44%) vs 29/119 (24%), p = 0.025) and the proportion with positive microscopy results (19/31 (61%) vs 28/90 (31%), p = 0.003) before initiation of treatment were significantly higher in the PR compared to the no PR group. Plaques (OR 5.12; 95% CI 2.26–11.61; *p*<0.001), oedematous (OR 4.23; 95% CI 1.43–12.5; *p* = 0.009) and category II lesions (OR 2.26; 95% CI 1.14–4.48; *p* = 0.02) were strongly associated with the occurrence of PR. The median time to complete healing (28 vs 13 weeks, p <0.001) was significantly longer in the PR group.

**Conclusions:**

Buruli ulcer patients who develop PR are characterized by high bacterial load in lesion samples taken at baseline and a higher rate of positive *M*. *ulcerans* culture. Occurrence of a PR was associated with delayed healing.

**Trial registration:**

ClinicalTrials.gov NCT02153034.

## Author summary

Buruli ulcer is a neglected tropical skin disease caused by the third most common pathogenic mycobacterium: *Mycobacterium ulcerans*. Paradoxical reaction, a phenomenon observed in some patients is characterised by worsening of existing lesion(s) with attendant pain and occurrence of new lesions during or after antibiotic therapy following an initial period of clinical improvement. This significantly affects treatment outcomes. In this clinical study, tissue samples obtained from patients were subjected to 16S rRNA/ IS*2404* qPCR to measure bacterial load. This was to identify a link between bacterial load in BU lesions and the development of paradoxical reactions following initiation of antibiotic treatment. We found that 13% of participants developed PR. Patients who developed PR had higher baseline bacterial load; a higher rate of positive *M*. *ulcerans* culture and persistently positive culture during antibiotic treatment. Occurrence of a paradoxical reaction was associated with delayed healing.

## Introduction

Buruli ulcer (BU) is a neglected tropical disease caused by infection with *Mycobacterium ulcerans* (*M*. *ulcerans*) which is common in rural parts of West African countries including Ghana. It causes large, disfiguring skin ulcers mainly in children aged 5 to 15 years although persons of any age can be affected [1]. Access to treatment in rural areas is limited and many patients present with late stage disease because of fear, suspicion about conventional medicine and the economic consequences for poor families [2]. The incidence of the disease is highly focal, and in Ghana for example, most cases occur in particular parts of the Ashanti Region [3]. The mode of transmission remains unknown but there have been major advances in understanding the mechanism of disease since the establishment of the WHO Buruli ulcer Initiative in 1998 together with improved diagnosis and management. The initial BU lesion is a subcutaneous painless nodule tethered to the skin or an intradermal plaque. These enlarge over a period of days to weeks and ulcerate in the centre. Ulcers are usually painless and have a necrotic base and irregular undermined edges [4, 5].

The mainstay of treatment is the combination of rifampicin and streptomycin or clarithromycin but additional treatment such as debridement and skin grafting, and early basic management with appropriate dressings and physiotherapy when an ulcer is close to a joint can minimize complications [6, 7]. The most common complication is paradoxical reaction occurring during or after treatment in 8–12%[5, 8] of patients in Africa. In an Australian population the phenomenon has been reported to occur more frequently (21%) in elderly patients [9]. Another study in Africa, also reported similar frequency of paradoxical reaction occurrence (22%) associated with trunk localization, larger lesions and genetic factors [10]. The time to development of paradoxical reaction varies widely between patients from antibiotic initiation, from few weeks in some patients to several months in others [5, 8, 9].

Paradoxical reactions cause anxiety to both patient and carer with the possibility that it represents uncontrolled or recurrent infection and indeed it is likely that earlier perceptions that antibiotics were ineffective for management of Buruli ulcer may have been influenced by such reactions. Culture of samples from the lesions are usually negative if the reaction occurs after completion of antibiotics but this does not exclude persistent infection since the sensitivity of culture for Mu is only 35–60%[11, 12]. Paradoxical reaction is thought to be due to an immunological response to residual *M*. *ulcerans* antigens which are known to persist for many months after successful treatment[9]. The immunological mechanism underpinning paradoxical reactions requires clearer elucidation in order to design appropriate evidence-based interventions for this important clinical phenomenon. Even though, several studies have associated paradoxical reactions with larger lesions, its relation to bacterial load has not been demonstrated. The aim of the present study was to investigate the clinical forms of paradoxical reactions in relation to their time of occurrence, the lesion type and bacterial load as potential risk factors for their occurrence.

## Methods

### Patients

From June 2013 to June 2017, patients with clinically suspected Buruli ulcer were screened at Agogo Presbyterian hospital, Tepa, Dunkwa and Nkawie-Toase Government hospitals. The diagnosis of Buruli ulcer was confirmed by *M*. *ulcerans* IS*2404* PCR. Patients who had already started antibiotic therapy or refused to participate were excluded. All categories of BU lesions were included.

### Study procedures

Demographic data of all participants, details of the timing and nature of paradoxical reactions were collected prospectively using WHO BU01 and study designed laboratory forms. The dimensions of lesions were documented using Silhouette (ARANZ Medical, Christchurch, New Zealand), a 3-dimensional imaging and documentation system together with digital photographs[13]. Patients were reviewed by an experienced clinician every 2 weeks up to 8 weeks and thereafter every month up to one year after completion of treatment. The time of complete healing was documented for all patients. All the patients recruited into the study were given combination antibiotic therapy of either rifampicin and clarithromycin or rifampicin and streptomycin for 56 days as recommended [7]. Two fine needle aspirates (FNA) were taken from non-ulcerated lesions; for patients with ulcerated lesions, two swabs from the undermined edges of ulcers were taken to confirm the diagnosis of BU by microscopy and PCR. The presence of viable bacteria was determined by taking samples for culture and 16S rRNA reverse transcriptase/IS*2404* qPCR assay. Samples were collected at baseline and at weeks 4, 8, 12 and 16 (only if lesions remained unhealed). When a paradoxical reaction occurred, samples were taken from those lesions for culture and 16S rRNA reverse transcriptase/ IS*2404* qPCR assay. Paradoxical reactions were defined by the presence of one or both of the following features as previously described: (i) an initial improvement in the clinical appearance of an *M*. *ulcerans* lesion during or after antibiotic treatment, followed by an episode of new inflammation, with or without pus formation, with significant enlargement of a healing lesion or its surrounding tissues or (ii) the appearance of a new lesion(s)[14].

Clinical samples were transported to the laboratory in appropriate transport media and processed immediately upon arrival at the laboratory. All routine laboratory tests and molecular assays were conducted at Kumasi Centre for Collaborative Research in Tropical Medicine (KCCR).

### Routine laboratory confirmation

For laboratory confirmation of Buruli ulcer disease, smear microscopy for acid-fast bacilli, culture on Lowenstein-Jensen medium and IS*2404* qPCR were performed by well-established methods as previously described[15–17]. A final diagnosis of Buruli ulcer was based on the IS*2404* qPCR result which was the most sensitive test.

### Combined 16S rRNA reverse transcriptase / IS*2404* qPCR assay

FNA and swab samples were transported from the study site to the KCCR laboratory stabilized in 500 μl RNA protect (Qiagen, UK). Whole transcriptome RNA and whole genome DNA were extracted separately from the same clinical sample. The RNA and DNA isolation was carried out within 5 hours of sample collection using the AllPrep DNA/RNA Micro kit (Qiagen, UK). RNA extracts were reverse transcribed into cDNA using Quantitect kit as described elsewhere[13]. The cDNA prepared was subjected to qPCR for detection of human glyceraldehyde-3-phosphate dehydrogenase (GAPDH) mRNA [17]. The detection of the GAPDH was for quality assurance purposes to confirm correct sample collection and to exclude false negative 16S rRNA RT qPCR results. All whole transcriptome RNA extracts from Buruli ulcer lesions tested positive when subjected to GAPDH mRNA RT qPCR at baseline.

The cDNA was then subjected to 16S rRNA qPCR and DNA to IS*2404* qPCR to increase the specificity for *M*. *ulcerans* and for quantification of the bacterial load as previously described[16]. Ten-fold serial dilutions of known amounts of a plasmid standard of IS*2404* (99 bp) and 16S rRNA (147 bp) (Eurofins MWG Operon, Ebersberg, Germany) were included with PCR amplification for preparation of a standard curve. *M*. *ulcerans* bacillary loads in original clinical samples were calculated based on threshold cycle values per template of IS*2404* qPCR (standard curve method) adjusted to the whole amount of DNA extract and the known copy number of 207 IS*2404* copies per *M*. *ulcerans* genome on average.

### Statistical analysis

The raw data generated from the study were entered in Microsoft Excel (Microsoft Corporation, Redmond, WA) and analyzed using GraphPad Prism version 5.0 (GraphPad Software, Inc., La Jolla, CA) STATA statistical package (StataCorp). Continuous variables such as age, IS*2404* copies and *M*. *ulcerans* 16SrRNA copies were compared using the Mann-Whitney *U* test. Chi-square test was used to compare the frequencies of all categorical variables, except the location of lesions which was compared using the Fisher’s exact test.

The frequency and percentage of missing values for each variable were collected, analysed and reported. When there were missing values for the variables of interest/outcome, exclusion of observations with missing values for the variables of interest/outcome was considered. Highly incomplete covariates (>33% of observations missing) were excluded from analyses.

The Kaplan-Meier survival analysis was used to determine the effect of developing paradoxical reaction on time to healing. Simple proportions of positive AFB and culture among the paradoxical reaction and the non-paradoxical reaction participants were also calculated. Logistic regression was performed to assess incidence rates and association of variables with PR. Univariate analysis was done to determine crude rate ratios and a multivariate analysis performed adjusting for age, gender and location of lesion to test associations with characteristics assessed at pretreatment. *P* value < 0.05 was considered statistically significant in all the analyses.

### Ethics statement

Verbal and written informed consent was obtained from all eligible participants and from parents or legal representatives of participants aged 18 years or younger. Ethical approval was obtained from the Committee of Human Research Publication and Ethics, School of Medical Sciences, Kwame Nkrumah University of Science and Technology, Kumasi, Ghana (CHRPE/AP/229/12) and registered with ClinicalTrials.gov identifier NCT02153034.

## Results

### Relationship between lesion type and form of paradoxical reactions

**Fig 1:**
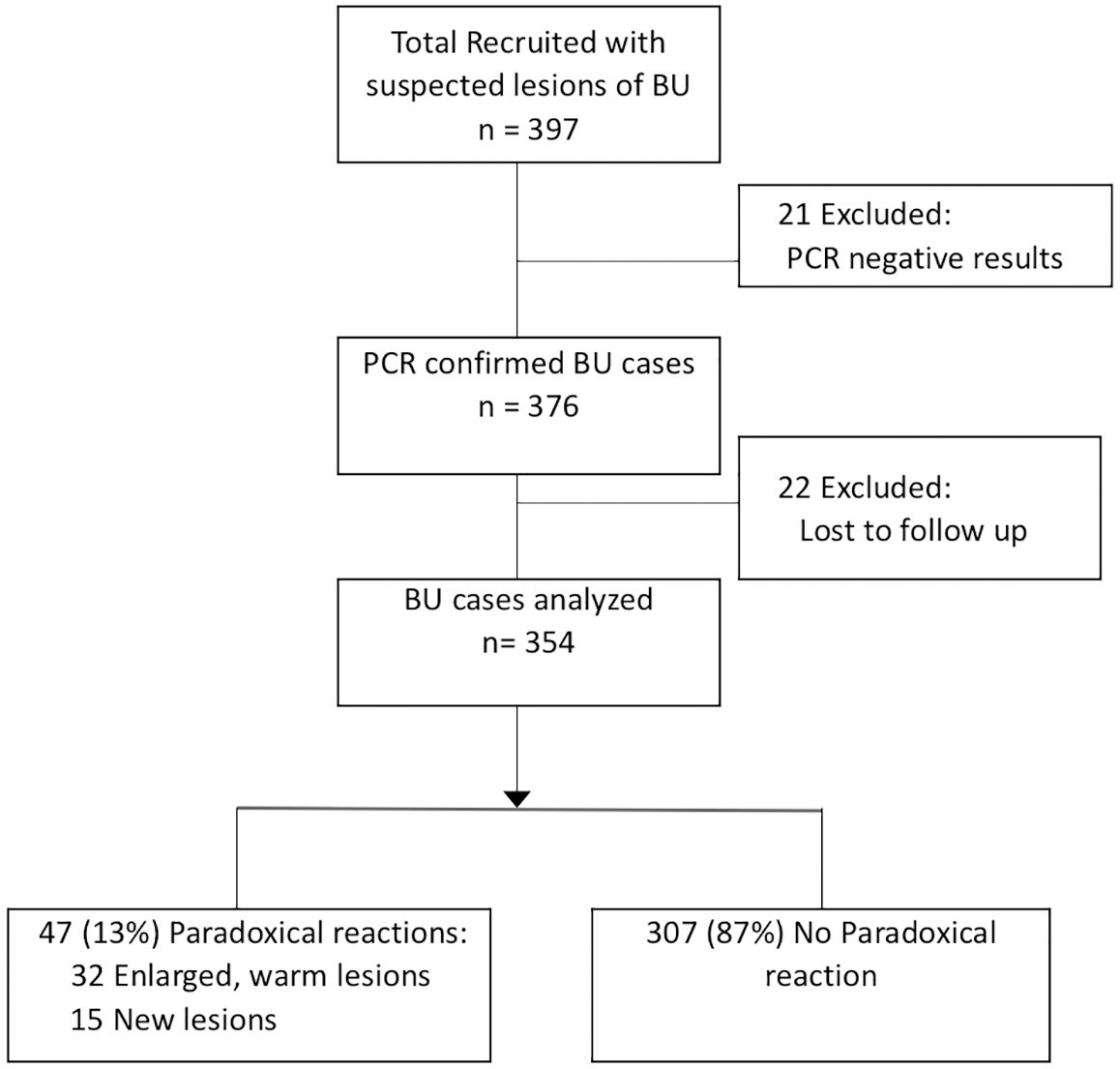
Flowchart of study participants, 2013–2017.

**Fig 2:**
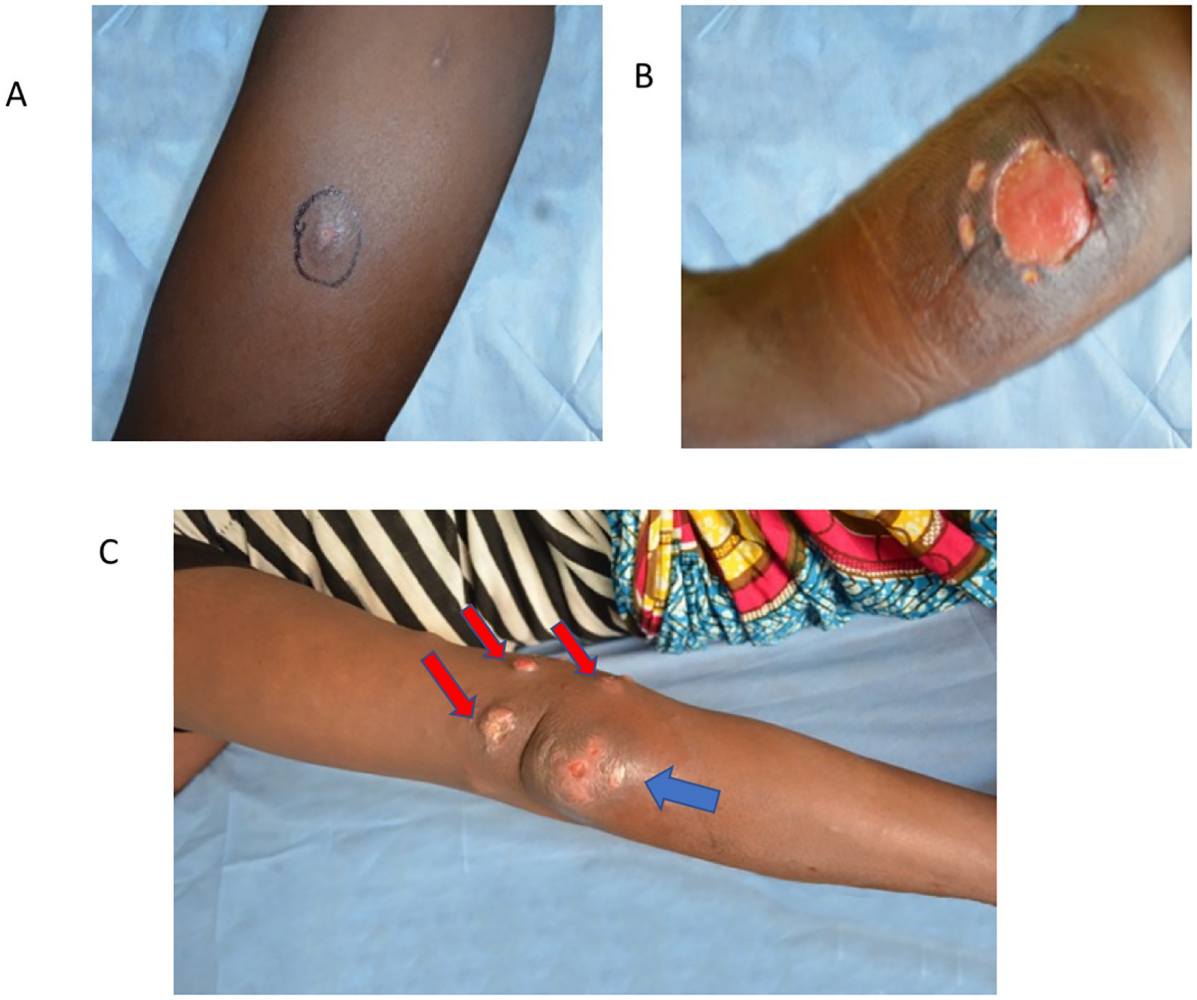
Typical paradoxical reactions in Buruli ulcer following antibiotic treatment. A: New nodular lesion on the left thigh of a patient with an indurated lesion before antibiotic treatment, B: Paradoxically enlarged ulcer of lesion A during antibiotic therapy at week 4. C: Multiple new lesions (red arrows) around the original lesion on the left elbow (blue arrow) at week 5 of antibiotic treatment.

A flowchart indicating the recruitment of patients is shown as Fig 1. Forty-seven (13%) out of 354 patients in the study developed paradoxical reactions. Of the 32 lesions that enlarged, 24 (51%) were just warm and enlarged, and 8 (17%) were also warm and pus filled, with or without pain. When enlargement happened, it was always during antibiotic therapy. Fifteen patients (32%) developed new lesion(s): 14 of them had a new lesion developing close to the original site and one had multiple new lesions around the existing one (Fig 2). Thirty-two (68%) paradoxical reactions occurred during antibiotic treatment. The median (IQR) time from the start of antibiotic administration to development of paradoxical reaction was 6 (4–11) weeks but the time to occurrence of an enlarged lesion (6; 4–8 weeks) was shorter compared to that for a new lesion (10; 5–28 weeks: p<0.01). A higher proportion (26/29 = 90%) of patients with nodules and plaques with paradoxical reaction had an enlarged lesion in comparison to patients with oedematous lesions and ulcers (6/18 = 33%: p<0.001). The majority of paradoxical reactions manifested as new lesions occurred in patients with oedematous lesions and ulcers (12/18 = 66%). No other variables significantly influenced the type of reaction that occurred.

### Association of paradoxical reaction with baseline *M*. *ulcerans* bacterial load

**Fig 3:**
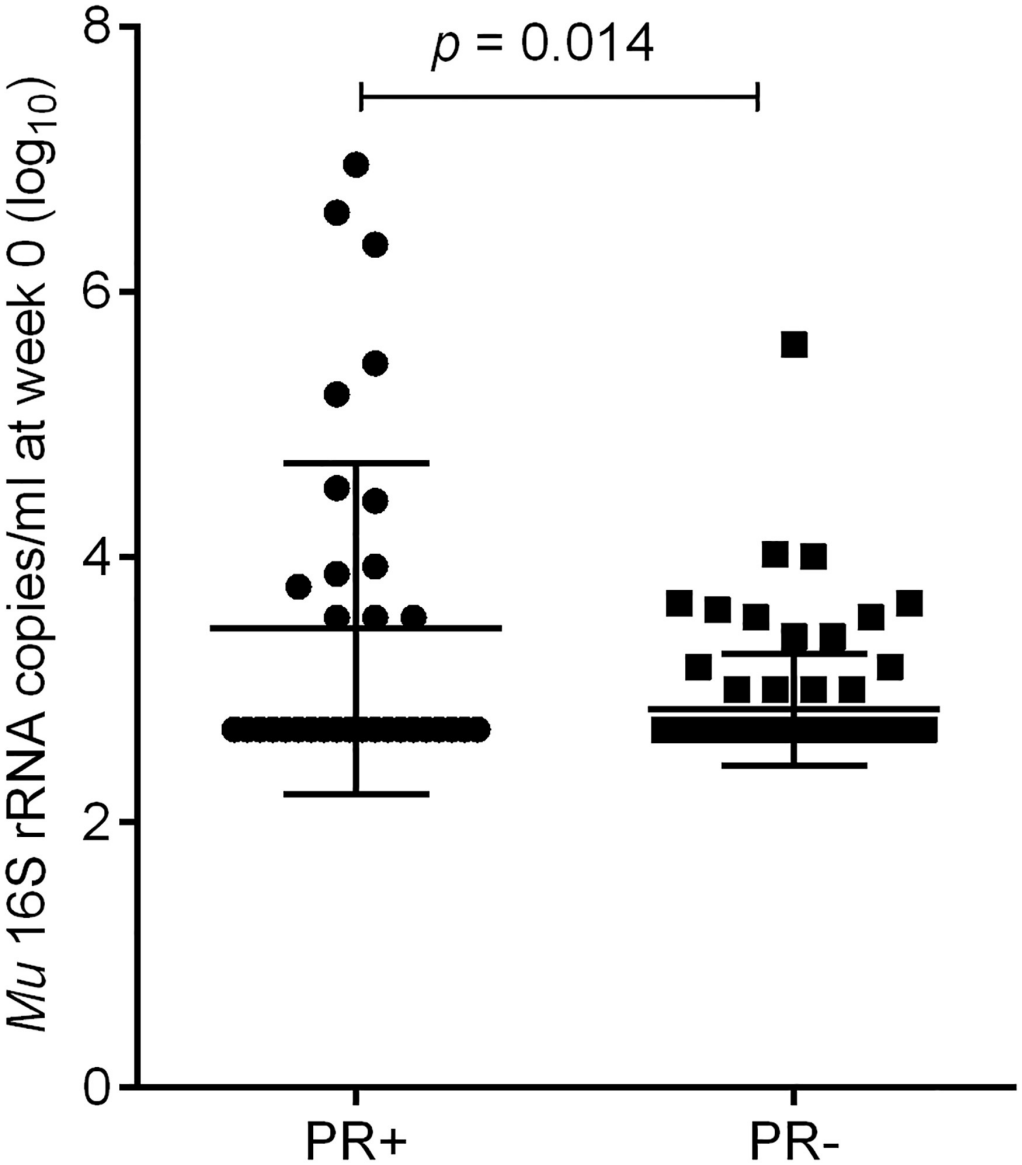
Increased bacterial load at baseline in patients who developed paradoxical reaction (PR+) in comparison to those who did not (PR-). Higher viable *M*. *ulcerans* in lesions of patients who subsequently developed paradoxical reaction post treatment as measured by 16S rRNA at baseline compared those who did not. Each point represents the number of copies per milliliter (log_10_) of sample. The horizontal bars show the means and standard deviations of each group.

Patients who developed a paradoxical reaction had a significantly higher bacterial load both in terms of IS*2404* copy numbers, median cps/ml (IQR) [500 (500–8000) vs 500 (500–500), *p* = 0.020] and higher viable organisms measured by *Mu* 16SrRNA, median cps/ml (IQR) [500 (500–4875) vs 500 (0–1000), *p* = 0.014] at baseline than patients with no paradoxical reaction (Fig 3). This was supported by the finding that a larger proportion of patients who developed a paradoxical reaction had positive AFB microscopy (61%) compared to those who did not (31%; *p* = 0.003). Similarly, the proportion of patients with positive culture results was significantly higher in those who developed paradoxical reaction (44% vs 24%; *p* = 0.025) (Table 1).

**Table 1:** Comparison of baseline characteristics of study participants between those who developed paradoxical reaction and those without paradoxical reaction.

Characteristic before treatment	Developed Paradoxical reaction		
	Yes (n = 47)	No (n = 307)	P value
Age, median years (IQR)	13 (9, 29)	16 (9, 35)	0.284
Gender, male, n (%)	28 (59.6)	150 (48.9)	0.171
Weight, median Kg (IQR)	32 (24, 52)	45 (26, 58)	0.058
Duration before seeking treatment, median weeks (IQR)	4 (2, 8)	4 (2, 8)	0.558
**Clinical Forms n (%)**			
Ulcer	12 (25.5)	169 (55.1)	**<0.0001**
Nodule	13 (27.7)	74 (24.1)	
Plaque	16 (34.0)	44 (14.3)	
Oedema	6 (12.8)	20 (6.5)	
**WHO Categories, n (%)**			
I (< = 5cm)	15 (31.9)	145 (47.2)	**0.042**
II (5-15cm)	26 (55.3)	111 (36.2)	
III (>15cm)	6 (12.8)	51 (16.6)	
**Location of lesion, n (%)**			
Lower limb (LL)	23 (48.9)	176 (57.3)	0.255*
Upper limb (UL)	22 (46.8)	107 (34.9)	
Other locations	2 (4.3)	24 (7.8)	
**Treatment type, n (%)**			
SR8	24 (51.1)	163 (53.1)	0.795
CR8	23 (48.9)	144 (46.9)	
Microscopy, AFBs positivity, ratio (%)	19/31 (61.3)	28/90 (31.1)	**0.003**
Bacteria Culture, confirm growth, ratio (%)	15/34 (44.1)	29/119 (24.4)	**0.025**
IS*2404*, median cps/ml (IQR)	500 (500–8000)	500 (500–500)	**0.020**
*Mu* 16SrRNA, median cps/ml (IQR)	500 (500–4875)	500 (0–1000)	**0.014**

Continuous variables were compared using the Mann-Whitney *U* test. All frequencies were compared using chi-square test except *location of lesion which was compared using the Fisher’s exact test. Abbreviations: WHO, World Health Organization; cps, copies; IQR, interquartile range; AFBs, Acid-fast bacilli; SR8, streptomycin-rifampicin for 8 weeks; CR8, clarithromycin-rifampicin for 8 weeks

### Characteristics of participants and associations with paradoxical reaction

Paradoxical reactions were related to the clinical form of the initial lesion. They were more common in patients with a plaque (27%) or oedematous lesion (23%) than in those with nodule (15%) or ulcer (7%) *(p* <0.001). Their incidence was significantly related to lesion category at presentation; 9% in category I and 10% in category III had a paradoxical reaction compared to 19% in category II (*p* = 0.04) (Table 2). Paradoxical reaction was equally common in patients who received streptomycin or clarithromycin combined with rifampicin. Using a logistic regression model, multivariate analysis showed that plaque (OR 5.42; 95% CI 2.25–13.04); *p*<0.001), oedematous lesion (OR 4.13; 95% CI 1.37–12.42; *p* = 0.012), nodular lesion (OR 2.63; 95% CI 1.12–6.17; p = 0.026) and category II lesions (OR 2.37; 95% CI 1.19–4.71; *p* = 0.014) were strongly associated with development of paradoxical reaction adjusting for age, gender and location of lesion.

**Table 2:** Association between baseline characteristics and occurrence of paradoxical reaction.

Baseline characteristics	Number (%) in Cohort	Number (%) developed PR	Unadjusted		Adjusted	
			OR (95% CI)	P value	OR (95% CI)	P value
**Age (years)**						
0–15	178 (50.3)	28 (15.7)	1.42 (0.75–2.68)	0.279	0.76 (0.24–2.38)	0.632
16–< 60	155 (43.8)	18 (11.6)	1		1	
≥ 60	21 (5.9)	1 (4.8)	0.38 (0.05–3.01)	0.36	0.45 (0.05–3.79)	0.395
**Gender**						
Male	178 (50.3)	28 (15.7)	1		1	
Female	176 (49.7)	19 (10.8)	0.65 (0.35–1.21)	0.173	0.67 (0.35–1.28)	0.226
**Weight (kg)**						
⁤ 40	147 (45.9)	28 (19.0)	1		1	
>40	173 (54.1)	19 (11.0)	0.52 (0.28–0.98)	0.045	0.57 (0.23–1.410	0.228
**Clinical Forms n (%)**						
Ulcer	181 (51.1)	12 (6.6)	1			
Nodule	87 (24.6)	13 (14.9)	2.47 (1.08–5.68)	**0.033**	2.63 (1.12–6.17)	**0.026**
Plaque	60 (16.9)	16 (26.7)	5.12 (2.26–11.61)	**<0.001**	5.42 (2.25–13.04)	**<0.001**
Oedema	26 (7.4)	6 (23.1)	4.23 (1.43–12.49)	**0.009**	4.13 (1.37–12.42)	**0.012**
**WHO Categories, n (%)**						
I (< = 5cm)	160 (45.2)	15 (9.4)	1		1	
II (5-15cm)	137 (38.7)	26 (19.0)	2.26 (1.14–4.48)	**0.019**	2.37 (1.19–4.71)	**0.014**
III (>15cm)	57 (16.1)	6 (10.5)	1.14 (0.42–3.09)	0.801	1.13 (0.41–3.10)	0.816
**Microscopy, AFBs present**						
No	74 (61.2)	12 (16.2)	1		1	
Yes	47 (38.8)	19 (40.4)	3.51 (1.50–8.20)	**0.004**	3.26 (1.36–7.86)	**0.008**
**Bacteria culture, confirm growth**						
No	109 (71.2)	19 (17.4)	1		1	
Yes	44 (28.8)	15 (34.1)	2.45 (1.11–5.43)	**0.027**	2.46 (1.07–5.63)	**0.033**
**Treatment type, n (%)**						
SR8	187 (52.8)	24 (12.8)	1		1	
CR8	167 (47.2)	23 (13.8)	1.08 (0.59–2.01)	0.795	1.16 (0.62–2.19)	0.641

Logistic regression model was used to test associations with characteristics assessed at pretreatment. Adjustment was performed for all characteristics presented. Abbreviations: WHO, World Health Organization; OR, odds ratio; CI, confidence interval; AFBs, Acid-fast bacilli

### Association of bacterial killing with paradoxical reactions

**Fig 4:**
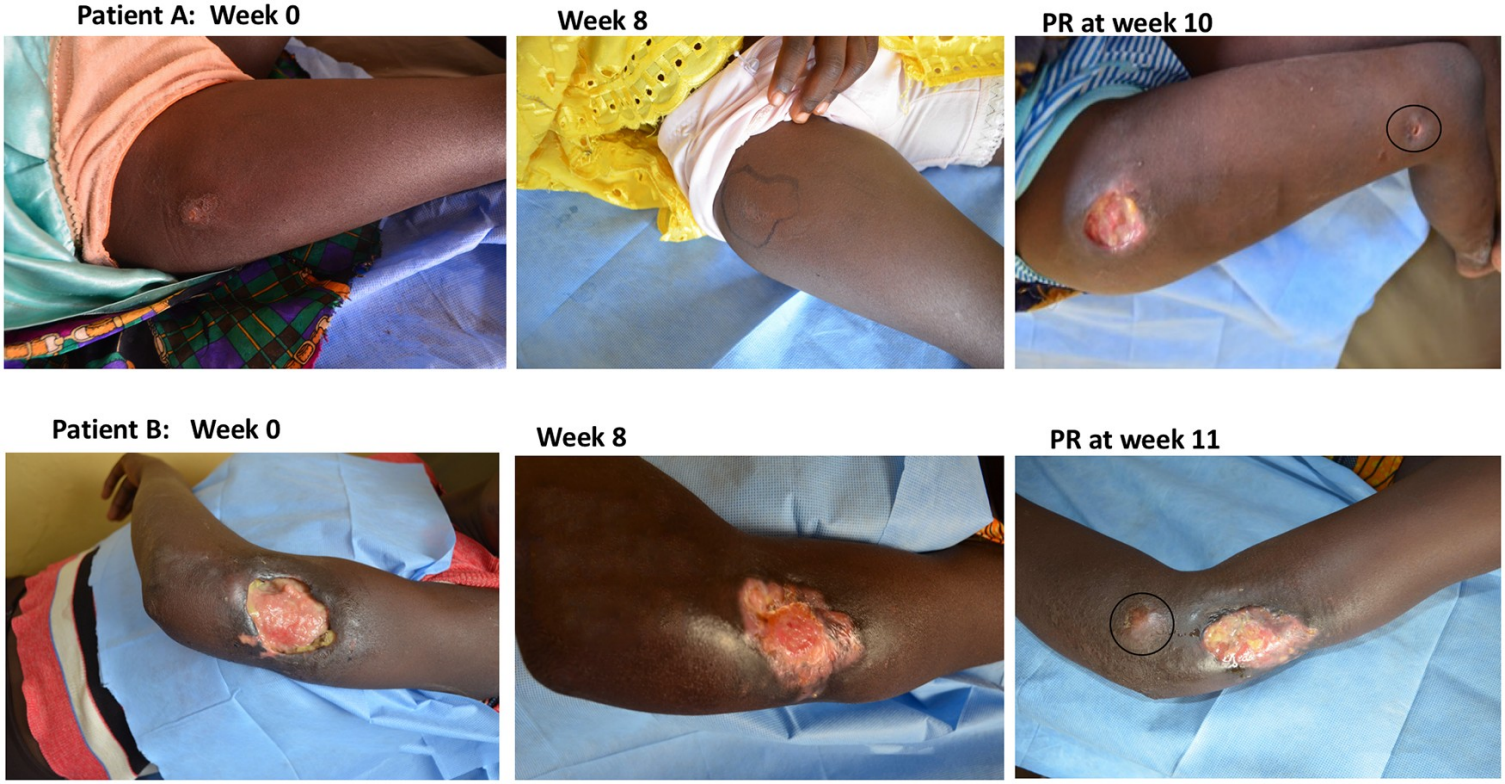
Images of 2 patients (A and B) who had positive 16S rRNA and culture results after 8 weeks of antibiotic treatment. Patient A; Presented with a plaque on the right thigh with surrounding induration (week 0) which reduced significantly after antibiotic treatment (week 8). A new lesion appeared on the right knee at week 10 at the same time as the original lesion on the right thigh re-ulcerated (PR at week 10). Patient B; Presented with an ulcer on the left elbow (week 0), there was improvement after antibiotic treatment as evidenced by formation of clean granulation tissue with some epithelization (week 8). A new lesion was seen close to the initial lesion (PR at week 11).

Positive cultures for *M*. *ulcerans* and/or positive 16S rRNA results were found in two out of fifteen patients (A and B in Fig 4) who had a paradoxical reaction after completion of antibiotic treatment. In patient A, a new lesion appeared on the right knee at week 10 at the same time as the original lesion on the right thigh re-ulcerated. Culture was positive from the new lesion. No additional antibiotics were administered and both lesions healed by week 24. Patient B, a 16-year-old girl with an ulcer on the left upper arm, developed a new lesion close to the initial lesion at week 11 which tested positive to combined 16S rRNA/IS*2404* qPCR assay. Both lesions healed completely at week 20 without further antibiotic therapy. All other paradoxical reactions before or after treatment had negative culture and 16S rRNA/IS*2404* qPCR results. In 23 patients who had positive *M*. *ulcerans* 16S rRNA or culture results at week 4, the median time to developing a PR was 6 weeks (IQR 4–8) compared with 13 weeks (IQR 6–26, p = 0.015) in 10 patients with negative week 4 *M*. *ulcerans* 16s rRNA/culture results.

### Outcome for patients with or without paradoxical reaction

**Fig 5:**
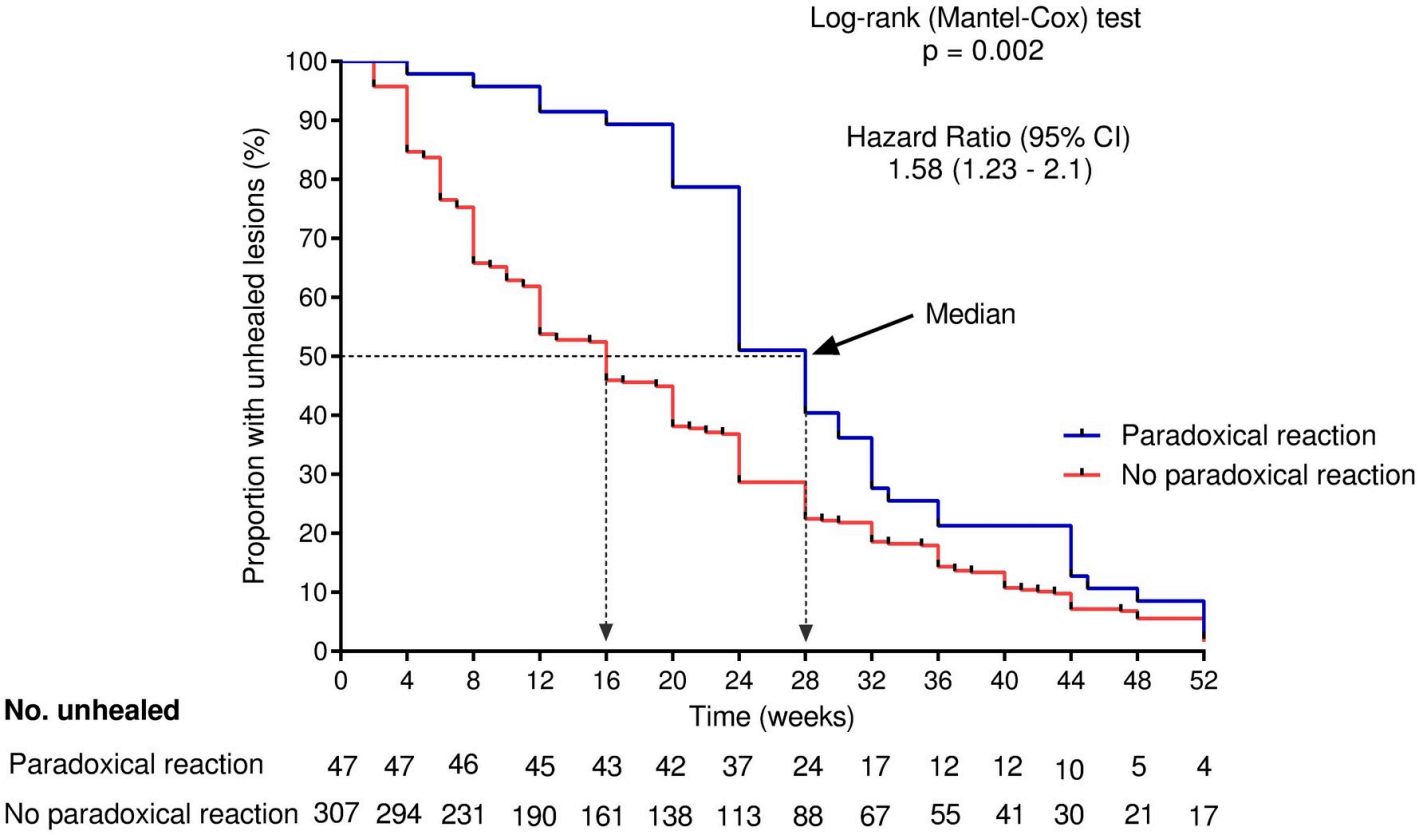
Kaplan Meier analysis for time to complete healing in Buruli ulcer patients who developed paradoxical reaction and those who did not following antibiotic treatment. Broken lines indicate the median healing time (weeks) for each group.

The median time to complete healing for patients with paradoxical reaction was 28 weeks compared to 16 weeks for those with no PR (p <0.001) (Fig 5). By the end of antibiotic treatment at 8 weeks only 2 of 47 (4%) PR group patients had completely healed compared to 42 of 307 (12%) in the no PR group. Patients with PR had a 1.58-fold increase (95% CI 1.23–2.10) in the time to complete healing of Buruli ulcer lesions compared to those who did not develop PR.

## Discussion

Paradoxical reactions have been reported previously in 8–12% of Buruli ulcer patients in Africa during or after treatment with antibiotics [5, 8]. In the present study the overall incidence was similar at 13% but lower than that which was reported in an Australian cohort [9], possibly because of the younger age distribution in this study compared to the elderly population in the Australian study; older age is a known risk of developing PR. We also recognised some distinctions in the clinical presentation. During antibiotic treatment for 8 weeks the common form of paradoxical reaction was re-enlargement of the lesion after healing had begun. This occurred more than 4 weeks after initiation of treatment by which time the necrotic tissue around the lesion had cleared by auto-debridement and it was usually associated with new inflammation, sometimes severe with pus formation. After completion of antibiotic treatment, paradoxical reactions consisted mainly of new inflammatory lesions adjacent to the original one, with or without new ulceration in the original lesion. This was more common when the initial lesion was an ulcer or oedematous lesion, possibly because bacteria were more widely disseminated around such lesion. It takes a longer time to kill the bacteria and clear mycolactone in the skin before inflammatory process due to dead bacteria in the skin starts which results in delayed PR even at distant sites occurring as new lesions.

It is difficult to make a distinction between paradoxical reaction and treatment failure when viable *M*. *ulcerans* can still be detected by culture or by the 16S rRNA assay in lesions during or after antibiotic treatment [13]. This was the case in 2 of 11 patients who developed new inflammatory lesions after completion of antibiotics but they were included as paradoxical reactions because they had clinical features of inflammation such as pain and/or pus formation and in each case the inflammation settled without further antibiotic treatment or any other additional therapy. The diagnosis of paradoxical reactions is usually clinical but investigations such as AFBs, mycobacterial culture, PCR for IS2404 repeat sequence and histopathology may be done. Viable organisms may also be detected using the 16S rRNA assay. In the present study, 2 patients were culture and 16S rRNA positive at the time of development of PR. Other studies have reported negative mycobacteria cultures at the diagnosis of PR [5, 9, 18]. In our study, no additional treatment (antibiotics, surgery or steroids) was given when PR was detected. However, other treatments including aspiration of pus without additional antibiotics[18], surgical excision [5] and administration of steroids [9] have been reported.

It is impossible to estimate accurately the total *M*. *ulcerans* bacterial load in a Buruli ulcer lesion but using a simple sampling method and estimating bacterial load by the number of copies of IS*2404* or 16S rRNA, we found an association between paradoxical reaction and high baseline bacterial load. This was supported by the finding that AFB detection and *M*. *ulcerans* culture were also more likely to be positive in these patients. The pathogenesis of paradoxical reaction in *M*. *ulcerans* disease is unknown but one hypothesis is that it is caused by an inflammatory reaction that, prior to antibiotic treatment, is suppressed by mycolactone, the *M*. *ulcerans* toxin. As the organisms are killed by antibiotics, mycolactone production ceases and its suppressive effect is lost causing a rebound of inflammation. This would be analogous to paradoxical reactions in *M*. *tuberculosis* and HIV co-infected patients when anti-retroviral treatment restores the immune response. In immune reconstitution inflammatory syndrome (IRIS) associated with HIV and *M*. *tuberculosis* or cryptococcal co-infection it has been postulated that antigens of the co-infecting pathogen accumulate before the immune response recovers leading to an excessive acute inflammatory response during anti-retroviral treatment[19].

There is a considerable disparity in the time to development of the paradoxical reaction. In this study it occurred from 4 to 28 weeks following initiation of therapy but 2 to 58 weeks is reported in other studies[9, 20]. We predicted that a paradoxical response would happen earlier in patients in whom *M*. *ulcerans* was cleared rapidly from their lesion. In fact, the opposite was the case; when the *M*. *ulcerans* 16S rRNA assay and culture were negative at 4 weeks, the paradoxical reaction occurred later than in those whose tests were still positive at 4 weeks. Unfortunately, we were unable to measure mycolactone in this study so the observation remains difficult to explain. Further studies of the pattern of cytokine secretion during treatment may shed some light on the problem.

## Supporting information

S1 File STROBE checklist. (PDF)

S2 File Raw data. This data shows the demographical information and clinical presentation data of all the participants recruited for this study.
(XLSX)

S3 File Laboratory data form. The laboratory entry form was used to collected extra information that was not included in the standard WHO BU01 form at each study time points. This extra information included wound measurements, photo documentation and catalogue of samples collected.
(PDF)
