# Supplementary material for: Paradoxical reactions in Buruli ulcer after initiation of antibiotic therapy: Relationship to bacterial load
Source: PLoS Negl Trop Dis. 2019 Aug 26;13(8):e0007689. doi: 10.1371/journal.pntd.0007689 (PMC6709892; doi:10.1371/journal.pntd.0007689)
Supplement: S1 File — (PDF) [file pntd.0007689.s001.pdf]

STROBE Statement—Checklist

	Item No	Recommendation	Manuscript Section
**Title and abstract**	1	(*a*) Indicate the study's design with a commonly used term in the title or the abstract	Abstract paragraph 2, page 2
		(*b*) Provide in the abstract an informative and balanced summary of what was done and what was found	Abstract paragraph 2, 3, 4. Page 2
**Introduction**			
Background/rationale	2	Explain the scientific background and rationale for the investigation being reported	Introduction paragraph 2, 3. Page 4
Objectives	3	State specific objectives, including any pre-specified hypotheses	Introduction paragraph 4. Page 5
**Methods**			
Study design	4	Present key elements of study design early in the paper	Methods paragraph 2. Page 5
Setting	5	Describe the setting, locations, and relevant dates, including periods of recruitment, exposure, follow-up, and data collection	Methods paragraph 1, 2. Page 5
Participants	6	(*a*) Give the eligibility criteria, and the sources and methods of case ascertainment and control selection. Give the rationale for the choice of cases and controls	Methods paragraph 1. Page 5
		(*b*) For matched studies, give matching criteria and the number of controls per case	N/A
Variables	7	Clearly define all outcomes, exposures, predictors, potential confounders, and effect modifiers. Give diagnostic criteria, if applicable	Methods paragraph 1, 2, 3. Pages 5 & 6
Data sources/ measurement	8*	For each variable of interest, give sources of data and details of methods of assessment (measurement). Describe comparability of assessment methods if there is more than one group	Methods paragraph 2,3,4. Pages 6 & 7
Bias	9	Describe any efforts to address potential sources of bias	N/A
Study size	10	Explain how the study size was arrived at	N/A
Quantitative variables	11	Explain how quantitative variables were handled in the analyses. If applicable, describe which groupings were chosen and why	Method paragraph 5. Page 7
Statistical methods	12	(*a*) Describe all statistical methods, including those used to control for confounding	Method paragraph 5. Page 7
		(*b*) Describe any methods used to examine subgroups and interactions	Method paragraph 5. Page 7

		(*c*) Explain how missing data were addressed	
		(*d*) If applicable, explain how matching of cases and controls was addressed	N/A
		(*e*) Describe any sensitivity analyses	N/A
**Results**			
Participants	13*	(a) Report numbers of individuals at each stage of study—eg numbers potentially eligible, examined for eligibility, confirmed eligible, included in the study, completing follow-up, and analysed	Results paragraph 1. Page 8
		(b) Give reasons for non-participation at each stage	Figure 1
		(c) Consider use of a flow diagram	Figure 1
Descriptive data	14*	(a) Give characteristics of study participants (eg demographic, clinical, social) and information on exposures and potential confounders	Results, Table 3 Pages 15 & 16
		(b) Indicate number of participants with missing data for each variable of interest	N/A
Outcome data	15*	Report numbers in each exposure category, or summary measures of exposure	Results, Table 1. Page 9 & 10
Main results	16	(*a*) Give unadjusted estimates and, if applicable, confounder-adjusted estimates and their precision (eg, 95% confidence interval). Make clear which confounders were adjusted for and why they were included	Results paragraph 2, Table 2. Pages 12 & 13
		(*b*) Report category boundaries when continuous variables were categorized	Results paragraph 3. Page 17
		(*c*) If relevant, consider translating estimates of relative risk into absolute risk for a meaningful time period	N/A

Other analyses	17	Report other analyses done—eg analyses of subgroups and interactions, and sensitivity analyses	Results paragraph 4. Page 18
**Discussion**			
Key results	18	Summarise key results with reference to study objectives	Discussion paragraph 1,2,3. Pages 18 & 19
Limitations	19	Discuss limitations of the study, taking into account sources of potential bias or imprecision. Discuss both direction and magnitude of any potential bias	Discussion paragraph 3. Page 19
Interpretation	20	Give a cautious overall interpretation of results considering objectives, limitations, multiplicity of analyses, results from similar studies, and other relevant evidence	Discussion paragraph 1, 3. Pages 18 & 19
Generalisability	21	Discuss the generalisability (external validity) of the study results	Discussion paragraph 4.

			Page 20
**Other information**			
Funding	22	Give the source of funding and the role of the funders for the present study and, if applicable, for the original study on which the present article is based	Page 20
