# Supplementary material for: Paradoxical reactions in Buruli ulcer after initiation of antibiotic therapy: Relationship to bacterial load
Source: PLoS Negl Trop Dis. 2019 Aug 26;13(8):e0007689. doi: 10.1371/journal.pntd.0007689 (PMC6709892; doi:10.1371/journal.pntd.0007689)
Supplement: S3 File — The laboratory entry form was used to collected extra information that was not included in the standard WHO BU01 form at each study time points. This extra information included wound measurements, photo documentation and catalogue of samples collected. (PDF) [file pntd.0007689.s003.pdf]

Burulipath study **Country: Ghana**

Laboratory Entry Form **Date** ……………20.…

Use separate form for each "visit" and each lesion (if multiple lesions) per patient!
Also fill WHO BU01 and BU04 for laboratory

A: Patient ID Number ………………………. **Hospital** ……………………….

Family name …………………………………. **First name** ……………………..

Village………………………………………. **District**…………………………

Age …………… **Sex** ☐ m ☐ f **BCG Scar** ☐ yes ☐ no

B: Classification **New case** ☐ **Relapse** ☐ **Follow up** ☐

C: Clinical Presentation

Nodule ☐ Papule ☐ Plaque ☐ Single lesion ☐
Ulcer ☐ Edema ☐ Osteomyelitis ☐ Multiple lesions ☐

D: Visit (Time of sample collection)

a) pre-treatment ☐ b) week 4 ☐ c) week 8 ☐ d) week 12 ☐ e) week 16 ☐

E: Duration of Disease

.... days .... weeks .... months

F: Location of the Lesion

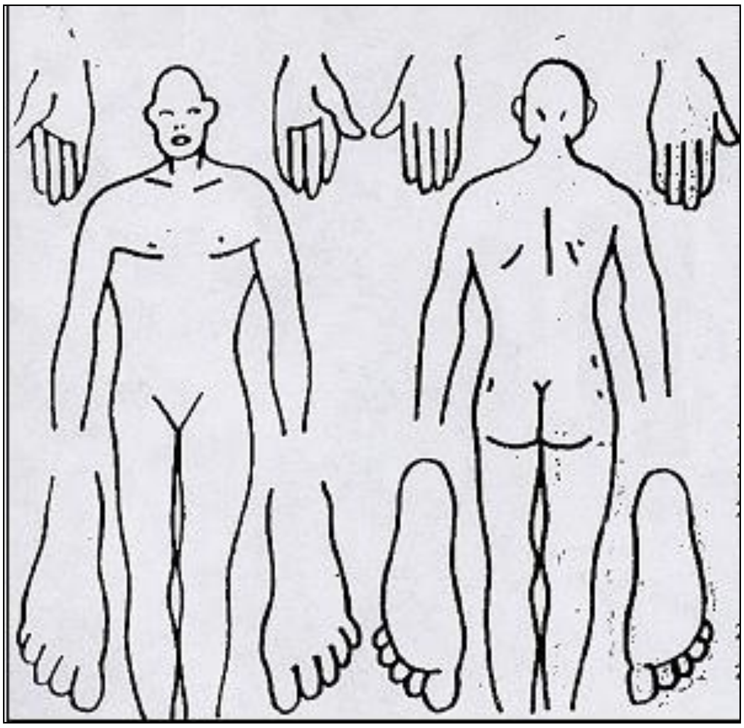

Remarks:

(Photographical) documentation: yes ☐ no ☐

Burulipath study **Country: Ghana**

lesion tracing/measured ☐ yes

G: lesion tracing

Indicate with 'H' when healed

**Week of visit**	**AREA (CM^2)**	**VOLUME(CM^3)**	**PERIMETER** (CM)	**MEAN. DEPTH (MM)**	**MIN. DEPTH** (MM)	**MAXI. DEPTH** (MM)	**LONG. DIAMETER** (CM)	**SHORT. DIAMETER** (CM)
**0**								
**2**								
**4**								
**6**								
**8**								
**12**								
**16**								
**20**								
**24**								
**28**								
**32**								
**40**								
**44**								
**48**								
**52**								

Burulipath study Country: Ghana

H: Clinical samples

**Baseline**	**FNA O**	**Swab O**	
PCR sample taken ☐			
**16S rRNA sample taken** ☐			
**Culture sample taken** ☐			
**AFB samples taken** ☐			
Blood taken ☐	**Heparinized blood O**	**EDTA blood O**	**Serum O**
**Week 4**	**FNA O**	**Swab O**	
**16S Rrna sample taken** ☐			
**Culture sample taken** ☐			
**AFB sample taken** ☐			
Blood taken ☐	**Heparinized blood O**	**3mm punch/swab O**	**Serum O**
**Week 8**	**FNA O**	**Swab O**	
**16S Rrna sample taken** ☐			
**Culture sample taken** ☐			
**AFB sample taken** ☐			
Blood taken ☐	**Heparinized blood O**	**3mm punch/swab O**	**Serum O**
**Week 12**	**FNA O**	**Swab O**	
**16S Rrna sample taken** ☐			
**Culture sample taken** ☐			
**AFB sample taken** ☐			
Blood taken ☐	**Heparinized blood O**		**Serum O**
**Week 16**	**FNA O**	**Swab O**	O
**16S Rrna sample taken** ☐			
**Culture sample taken** ☐			
**AFB sample taken** ☐			
Blood taken ☐	**Heparinized blood O**		**Serum O**
**Paradoxical reaction**			
Blood taken	**Heparinized blood O**		**Serum O**
	**FNA O**	**Swab O**	
**PCR samples taken** ☐			
**16S rRNA sample taken** ☐			
**Culture sample taken** ☐			
**AFB sample taken** ☐			

Burulipath study

Country: Ghana

Heparinized blood 6mls,
EDTA 2mls,
Serum 5 ml (whole blood)

I: Functional limitation assessment done

**Wk 0**	**Yes**	**No**
**Wk 8**	**Yes**	**No**
**Wk 52**	**Yes**	**No**
